# Fibroepithelial Polyp of the External Auditory Canal: A Case Report and a Literature Review

**DOI:** 10.1155/2013/818197

**Published:** 2013-05-29

**Authors:** Nobuaki Tanaka, Takeshi Matsunobu, Akihiro Shiotani

**Affiliations:** Department of Otolaryngology-Head and Neck Surgery, National Defense Medical College, 3-2 Namiki, Tokorozawa, Saitama 359-8513, Japan

## Abstract

This paper reports the first case of fibroepithelial polyp arising independently of the external auditory canal. A 16-year-old female patient presented to our clinic for aural fullness of the left side. Physical examination revealed a papillomatous tumor at the posterior wall of the inlet of the left external auditory canal. After biopsy, which yielded a diagnosis of benign papilloma, the patient underwent tumor excision. Final diagnosis was fibroepithelial polyp. One week after resection, aural fullness had resolved. Fibroepithelial polyp is a benign lesion and occurs mainly in the skin, ureteropelvic system, and genitals. In the head and neck area, there are reports on fibroepithelial polyp of the tongue, piriform fossa, inferior nasal turbinate, and tonsil, in addition to the skin, but none on independent fibroepithelial polyp of the external auditory canal. Excision of fibroepithelial polyp of the external auditory canal is advisable, especially in the presence of any symptoms, and should be preceded by confirmation of nonmalignancy by biopsy, if possible.

## 1. Introduction

Fibroepithelial polyp is a benign lesion of mesothelial origin and is one of the most common cutaneous lesions. It is generally an incidental finding on the skin of the neck, trunk, or face and is also known as fibroma or acrochordon, representing a nonspecific and benign growth pattern as opposed to a specified entity [1]. In addition to the skin, fibroepithelial polyp infrequently occurs in the ureteropelvic system, genitals, or bronchus [2–5]. Fibroepithelial polyp is benign and presents with an indolent clinical course. However, problems may arise depending on location, especially in the case of polyps in the bronchus that cause occlusion.

To the best of our knowledge, there is no report on fibroepithelial polyp arising independently of the external auditory canal. Although only one report about fibroepithelial polyp of the external auditory canal has been published, this was believed to be a reactive change in the skin overlying an osteoma [6]. Therefore, we present this very rare case and also review the literature of fibroepithelial polyp occurring independently in the head and neck area.

## 2. Case Report

A 16-year-old female Japanese patient was referred to our clinic by an otolaryngologist for suspected papilloma of the external auditory canal; she presented to our clinic with a 2-week history of aural fullness of the left side. She had no other medical problems except allergic rhinitis triggered by house dust, mites, and pollen from Japanese cedar and cypress trees.

**Figure 1:**
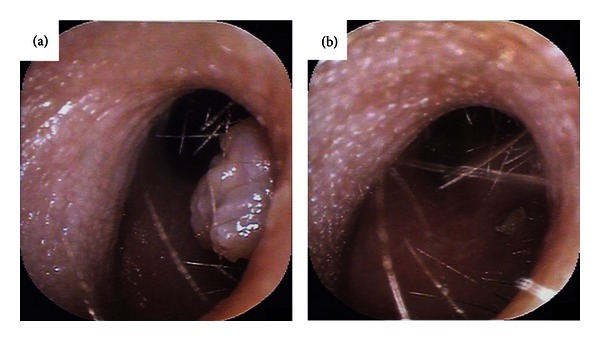
Endoscopic photographs of the left external auditory canal. (a) Fibroepithelial polyp at the posterior wall of the inlet of the left external auditory canal. (b) Left external auditory canal 1 week after resection of the fibroepithelial polyp.

On physical examination, a papillomatous tumor was observed at the posterior wall of the inlet of the left external auditory canal (Figure 1(a)). The rest of the ENT examination and the results of blood tests yielded normal findings. Biopsy of the tumor was performed with topical anesthesia on the same day, and a diagnosis of papilloma was received 2 days later. Because there was no evidence of malignancy and the tumor was small enough to be resected completely in a day surgery, the patient underwent en bloc excision of the tumor with topical and local anesthesia after we had obtained informed consent from her and her mother.

**Figure 2:**
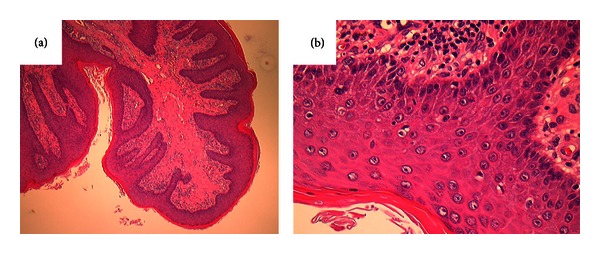
Fibroepithelial polyp covered by epidermis with hyperkeratosis leading to irregular epidermal projections and interstitial proliferation (hematoxylin-eosin staining: (a) ×40; (b) ×400).

Histopathological investigation revealed that the tumor was covered by epidermis with hyperkeratosis and had irregular epidermal projections and interstitial proliferation (Figure 2). Perivascular infiltration of lymphocytes or plasma cells was also found. Regarding the degree of papillary growth of this tumor, the exophytic growth pattern was of a lower level than that of papilloma. These findings suggested a diagnosis of fibroepithelial polyp.

One week after resection of the polyp, the postoperative wound at the external auditory canal had almost completely epithelialized (Figure 1(b)), and the chief complaint, aural fullness, had resolved. No obvious evidence of recurrence has been seen at the resected area for a postoperative follow-up period of 20 weeks.

## 3. Discussion

Fibroepithelial polyp is regarded as a pseudotumor caused by inflammation or hyperplasia secondary to local lesions. It is a benign lesion with an extremely low incidence of malignancy, and its etiology remains largely unknown [7]. As mentioned above, major sites of lesion formation include the skin, ureter, renal pelvis, genitals, and bronchus. Cases of fibroepithelial polyp occurring independently in the head and neck area are rare, and the sites include the oropharynx, tongue, and inferior nasal turbinate, in addition to the skin [7–9].

Table 1 shows a summary of the literature, to our knowledge, of fibroepithelial polyp occurring independently in the head and neck area (excluding skin) since 2000. The findings of these reports reveal that fibroepithelial polyp arose from various sites in the head and neck area (ear, nasal cavity, oral cavity, oropharynx, and hypopharynx), and occurrence was not related to age or sex. Under local or general anesthesia, all 5 patients underwent total excision of the polyp, one of which was resected together with the tonsil. Preoperative biopsy was only performed in our case, but we recommend biopsy before total surgical resection, if possible, to confirm whether the polyp is benign or malignant. In the case of relatively large polyps in the laryngopharynx that might cause imminent airway compromise, Mangar et al. and Farboud et al. have advocated immediate resection of the polyp or airway management to avoid upper airway obstruction [7, 8].

Generally, tumor-like lesions of the external auditory canal, so-called “aural polyps,” include exostosis, osteoma, fibrous dysplasia, granuloma, ceruminous gland tumor, epidermoid cholesteatoma, papilloma, and malignancies [10, 11]. Diagnoses of inflammatory polyp, cholesteatoma, and polyp caused by an underlying ventilation tube, *Mycobacterium tuberculosis*, and Langerhans cell histiocytosis have also been reported in approximately 35 patients younger than 20 years of age [12]. In the report, 15 (43%) of 35 cases were diagnosed as inflammatory polyp associated with chronic otitis media, cholesteatoma was documented in 10 (29%) patients, and an underlying ventilation tube was the suspected cause of the polyp in 8 (23%) patients. The remaining 2 cases were *Mycobacterium tuberculosis* and Langerhans cell histiocytosis. Our patient was 16 years of age, and fibroepithelial polyp should be considered a rare differential diagnosis of “aural polyp” both in young and adult cases.

There is only one previous report on fibroepithelial polyp of the external auditory canal, and in this case, the polyp was caused by a reactive change in the skin overlying an osteoma [6]. The patient underwent surgical resection of the osteoma with fibroepithelial polyp and had previously undergone an aural polypectomy more than 12 years earlier, which had been reported histopathologically as an “aural inflammatory polyp covered by stratified squamous epithelium.” Therefore, it could not be denied that this fibroepithelial polyp had arisen secondarily, not independently, or in association with an initial inflammatory polyp. On the other hand, in our case, there were no clinical findings suggesting the presence of previous history of aural polyp or other ear disorders or mechanical stimulation of the external auditory canal such as overuse of an ear pick. Thus, this is the first report on independent fibroepithelial polyp of the external auditory canal.

Mass lesions that narrow or occlude the auditory canal can cause hearing loss, otitis externa with resultant otalgia and otorrhea, tinnitus, aural fullness, and vertigo [10, 13]. In this case, the patient experienced aural fullness on the affected side, and resection of the fibroepithelial polyp resolved the symptoms. A previous report also showed that ear symptoms assumed to be due to “aural polyp” could be alleviated by excision of the polyp [6].

Fibroepithelial polyp is a benign lesion and seldom undergoes malignant transformation. “Aural polyps,” however, should be resected to confirm the diagnosis even if no symptoms are described because an association between “aural polyp” and cholesteatoma and an external auditory canal polyp accompanying squamous cell carcinoma have been reported previously [14, 15]. In the case of squamous cell carcinoma of the external auditory canal, aggressive surgical resection such as lateral temporal bone resection is recommended to achieve a cure, even when the lesion is small [15].

We have reported a case of fibroepithelial polyp of the external auditory canal. It is advocated that “aural polyps” are resected for confirmation of diagnosis and possible resolution of ear symptoms, such as hearing loss, tinnitus, and aural fullness, after confirmation of nonmalignancy by biopsy.

## Figures and Tables

**Table 1:** Literature review of fibroepithelial polyp occurring independently in the head and neck area (excluding skin) since 2000.

Site of lesion formation	Age/sex	Biopsy before surgery	Anesthesia for surgery	Treatment	References
Tongue	42/M	−	Unknown	Excision	Lloyd et al., 2001 [1]
Piriform fossa	60/M	−	General	Excision	Mangar et al., 2004 [8]
Inferior nasal turbinate	69/F	−	General	Excision	Perić et al., 2009 [9]
Tonsil	33/M	−	Unknown	Tonsillectomy	Farboud et al., 2010 [7]
External auditory canal	16/F	+	Local	Excision	Tanaka

M: male; F: female.
